# A review of recent clinical trials to evaluate disease-modifying therapies in the treatment of cardiac amyloidosis

**DOI:** 10.3389/fmed.2024.1477988

**Published:** 2024-10-30

**Authors:** Sindhuja Senigarapu, James J. Driscoll

**Affiliations:** ^1^Case Comprehensive Cancer Center, Case Western Reserve University, Cleveland, OH, United States; ^2^Adult Hematologic Malignancies & Stem Cell Transplant Section, Seidman Cancer Center, University Hospitals Cleveland Medical Center, Cleveland, OH, United States; ^3^Division of Hematology and Oncology, Case Western Reserve University School of Medicine, Cleveland, OH, United States

**Keywords:** amyloidosis, plasma cell, light chain, transthyretin, proteasome

## Abstract

Cardiac amyloidosis (CA) is a serious condition that results in infiltrative cardiomyopathy and heart failure with preserved ejection fraction (HFpEF) that is caused by the extracellular deposition of amyloid fibrils within heart tissue. While many important features of CA have been known for years, its prevalence in elderly patients with HF is increasingly being recognized. Plasma cells produce monoclonal immunoglobulin light chains which results in the formation and aggregation of amyloid fibrils that are responsible for AL amyloidosis. CA is classified as originating from either transthyretin (ATTR) or light chain (AL) amyloidosis. ATTR CA may result from a genetic mutation in the *TTR* gene, which is inherited (ATTRv), or from age-related deposition from wild-type ATTR (ATTRwt). Cardiac involvement in AL amyloidosis is attributed to either of two mechanisms: the extracellular deposition of amyloid fibril in the myocardium, or direct cardiotoxicity from the fibril aggregates. Typing of amyloid fibrils, a critical determinant of therapy, has also improved with wider availability of laser capture and mass spectrometry of histologic specimens. Specific and accurate evaluation of CA is now possible using cardiac magnetic resonance imaging and bone scintigraphy tracers. Survival in CA has improved markedly as novel chemotherapy agents have become available, but challenges remain in advanced disease. Broadening the amyloid-specific therapeutic landscape to include RNA inhibitors, fibril formation stabilizers and inhibitors, and immunotherapeutic targeting of amyloid deposits holds promise and may improve outcomes in systemic and cardiac amyloidoses. Treatment strategies for CA has recently undergone transformative changes, leading to some progress in outcomes for certain patients. Here, we discuss the basic features of CA as well as the emergence of novel, disease-modifying strategies that have been recently evaluated in clinical trials for the treatment of CA.

## Introduction

Amyloidoses are a collection of systemic infiltrative diseases characterized by the extracellular deposition of insoluble proteins caused when the protein amyloid abnormally deposits into one or more organs (1–9). Such diseases are classified as either systemic or localized and further sub-classified according to the site of amyloid deposits and the site of precursor protein production (6–11). Amyloidoses comprise a group of diseases that are triggered by the misfolding of a soluble precursor protein (1–11). These diseases are relatively rare but represent global epidemics that remain incurable and are responsible for significant health, social and economic implications (4–12). Hence, there exists an urgent, unmet clinical need for translational research strategies to probe the origin of disease and pathological manifestations of amyloidosis. Such studies may foster the development of more effective, therapeutic strategies to improve patient quality-of-life and overall survival (OS) (13–21).

AL amyloidosis is the most prevalent disease variant and affects >10 people/ million/ year (4, 12). The age-specific incidence rate of AL amyloidosis increases with each decade over 40 years of age, with 64 being the median age at diagnosis (Table 1). Less than 5% of affected patients are <40 years of age. Similar to multiple myeloma (MM), affected males outnumber females 3:2. While the incidence of MM is 2-fold higher in Black than White patients, AL amyloidosis does not have an apparent ethnic specificity (22). AL amyloidosis occurs in association with MM, Waldenström macroglobulinemia (WM) and non-Hodgkin’s lymphoma (23). Approximately 10% of patients with AL amyloidosis may have MM (24, 25). Unlike MM, patients with AL amyloidosis usually have <20% involvement of the bone marrow with a plasma cell clone (13).

**TABLE 1:** Comparison of the key features of amyloid light chain (AL), systemic autoimmune or infection (AA); ATTR variant (ATTRv), and ATTR wildtype (ATTRwt).

Type of amyloid	Systemic amyloidosis		Transthyretin amyloidosis	
Subtype	AL	AA	ATTRv	ATTRwt
Protein deposited	Light	Amyloid A	Variant TTR mutants	Wildtype TTR monomers
Etiology	Plasma cell dyscrasia	Systemic autoimmune or infections	Familial inherited TTR mutations	Age-related, >75 years of age
Tissue affected	Kidney cardiac hepatic	Renal	Cardiac	Cardiac carpal tunnel
Sex	Male > Female 3:2	Male > Female	Male > > > Female	Male = Female
Presentation age	64 <5% are under 40	Variable	30’s and 50’s	Late 60’s
Genetics			V1221 common in African Americans	Male dominance Autosomal Dominant
Association with MM	10%			
Bone marrow involvement	<20%			
Median OS	1–3 years	1–3 years	2.5 years (Val122Ile)	3.6 years
Therapy	Chemotherapy ++/- ASCT	Treat the underlying condition	Supportive care Liver/ heart transplantation	Supportive care
Tafamidis	The first FDA-approved treatment for ATTR-CM			
Patisiran	A small interfering RNA, is FDA-approved to treat polyneuropathy in hereditary ATTR			
	Clinical trials- e.g., Inotersen, NI006			

Based upon information provided in references (4–12, 17, 26, 27, 113–120, 125).

Systemic amyloidosis due to accumulation of misfolded transthyretin (TTR) protein causes cardiomyopathy in approximately 50,000 to 150,000 people in the US (26–33). TTR, also known as prealbumin, is a circulating protein synthesized by the liver that transports thyroid hormone and the retinol (vitamin A)-binding protein 4 complex (26–30). TTR typically exists as a homotetramer, but TTR can dissociate into its constituent subunits that subsequently misfold and aggregate into amyloid fibrils. ATTR is the term used to refer to amyloidosis caused by misfolding of the TTR protein. ATTR amyloidosis manifests principally as cardiomyopathy and polyneuropathy (26–33). Cardiomyopathy due to ATTR amyloidosis can cause heart failure, arrhythmia, and death, while ATTR deposition in nerves causes a small-fiber peripheral and/or autonomic polyneuropathy. ATTR deposition in ligaments manifests as carpal tunnel syndrome and cervical or lumbar spinal stenosis (34–39). Inherited *TTR* gene variants cause amino acid substitutions promoting amyloidogenesis.

ATTR amyloidosis due to genetic variant(s) affects an unknown number of people. More than 130 gene variants in TTR have been identified. However, a common inherited pathological *TTR* variant exists in more than 1.5 million people in the US, but the proportion of variant carriers who develop clinical manifestations of amyloidosis is not well defined. ATTR due to a genetic variant manifests as either cardiomyopathy with or without coexistent polyneuropathy with a phenotype that depends on the specific variant. Point mutations, deletions, and premature stop codons result in structural changes that predispose to fibril formation and the development of hereditary amyloidosis (21, 30, 40–47). Depending upon the type of amyloidosis, factors affecting protein folding and stability, including molecular chaperones and failure of disaggregating pathways, also contribute to pathogenesis. The protein fibers in the amyloid light chain consist of monoclonal light chains. In the other form of ATTR amyloidosis, a normal (wildtype) *TTR* genotype (ATTRwt) is present without the presence of detectable, destabilizing *TTR* variants. The mechanisms underlying TTR protein dissociation and amyloid fibril formation are incompletely understood but ATTR amyloidosis is more common in older people. ATTRwt is the most common type of ATTR amyloidosis (∼ 75% of ATTR amyloidosis) and typically manifests as cardiomyopathy, and fewer than 10% of patients have concurrent polyneuropathy (48–54).

At least 60 heterogeneous amyloidogenic proteins that have been identified and ∼ 30 have been associated with human disease (17). A common feature of the amyloidogenic proteins is the tendency align as antiparallel β-pleated sheets. The sheets are arranged in rigid, non-branching fibrils that are deposited and mechanically disrupt or distort the heart, liver, kidneys, nervous system, and gastrointestinal tract. Protein misfolding leads to formation of oligomers, aggregates, and amyloid fibrils that are characterized by pleated β-sheets which are deposited in various organs and tissues. The fibrils deposited in extracellular tissues are composed of low molecular weight subunits of a variety of proteins, which typically range from 5 to 25 kilodaltons. Many of these proteins normally circulate as constituents of plasma (17, 38). Organ dysfunction in AL amyloidosis is related to precursor protein proteotoxicity and/or disruption of the architecture, direct cytotoxic effects from protein aggregates and oligomers, or both (2, 55–58). The result is progressive organ dysfunction, organ failure, and eventually death.

ATTRv (or ATTRvariant) slowly progressive peripheral sensorimotor and/or autonomic neuropathy (Table 1). Typically, sensory neuropathy initiates in the lower extremities with paresthesia and hypesthesia of the feet, followed by motor neuropathy. In those with early-onset disease, autonomic neuropathy is the first manifestation of the condition. Other symptoms include orthostatic hypotension, constipation alternating with diarrhea, nausea, vomiting, delayed gastric emptying, sexual impotence, anhidrosis, and urinary retention or incontinence. Amyloid deposits into extracellular space of the myocardium results in thickening and stiffening of ventricular walls with resultant heart failure and conductive dysfunction. Senile ATTR amyloidosis is similar to familial ATTR amyloidosis, except that the non-mutated TTR protein is deposited. Individuals typically only manifest the disease in their later years, typically at 65 years of age or beyond. Due to the gradual accumulation of amyloid deposits in this disease variant, the prognosis is generally more favorable compared to AL amyloidosis and familial ATTR amyloidosis.

**FIGURE 1:**
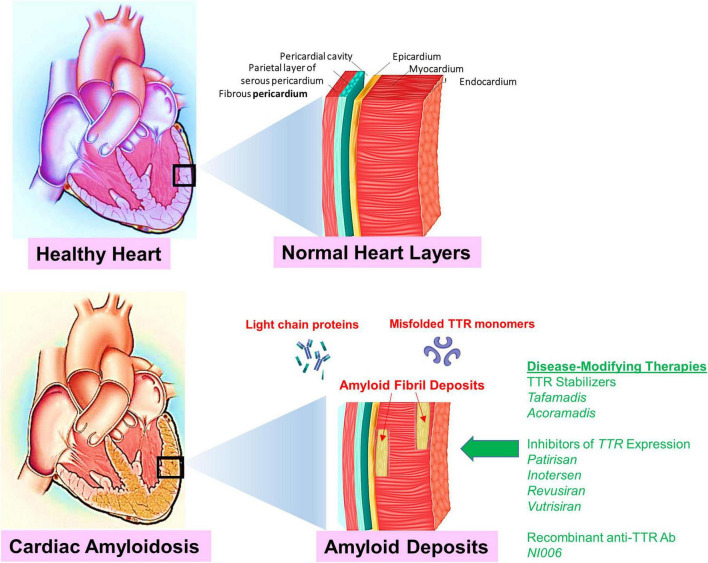
Shown is an illustration to compare normal healthy cardiac tissue to cardiac tissue infiltrated with amyloidosis deposits (red arrows). The accumulation of deposits result in a restrictive cardiomyopathy caused by extracellular deposition of proteins in the myocardium. Targets for disease-modifying therapies in cardiac amyloidosis include TTR silencing, TTR stabilization, and TTR disruption. Novel disease-modifying therapies are indicated (green arrow). Emerging novel disease-modifying therapies emphasize the necessity to diagnose CA at an early stage and to identify patients who may benefit from these potentially life-extending or life-saving therapies.

**Etiology.** Cardiac amyloidosis occurs due to the extracellular deposition of a toxic component called amyloid, which refers to an amalgam of abnormally folded proteins and other matrix-forming components such as proteoglycans, glycosaminoglycans, collagen, and laminin (Figure 1). The abnormally folded proteins derive from two sources: AL chain proteins and ATTR. Deposition of these fibrils in the extracellular space leads to the stiffening of the myocardium and depressed cardiac function. This deposition primarily affects diastolic function until late in the disease when left ventricular systolic function is also affected.

**Symptoms and presentation.** Symptoms of AL and ATTR amyloidosis are determined by the organs involved in the disease (1–5, 8). In cardiac amyloidosis, amyloid protein deposits in the heart muscle causes it to become thick, stiffened and weak over time (52, 59–61). Early signs include low voltage on EKG, concentric ventricular thickening on echocardiography, and diastolic dysfunction. Amyloid protein deposits in the heart can lead to cardiac symptoms such as congestive heart failure (CHF). Symptoms include shortness of breath during activity or while at rest, fatigue, fluid buildup in the abdomen and legs, and difficulty lying flat at night. Heart rhythm abnormalities are also present and symptoms include lightheadedness, dizziness, palpitations, shortness of breath, and fatigue. If amyloid deposits in the valves in the heart, this can lead to leaky (regurgitant) or narrowed (stenotic) valvular disease. ATTR amyloidosis has been found in some patients being treated for severe aortic stenosis. Non-cardiac symptoms or other differential diagnoses related to deposits in other parts of the body, include (bilateral) carpal tunnel syndrome, lumbar spinal stenosis; peripheral and autonomic neuropathy. Orthostatic hypotension, purple discoloration around eyelids, easy bruising and macroglossia are specific to AL amyloidosis.

**Diagnosis of cardiac amyloidosis.** Nonspecific symptoms linked to AL amyloidosis often contribute to diagnostic delays (60–67). Consideration of AL amyloidosis is crucial in patients with unexplained proteinuria, restrictive cardiomyopathy, peripheral neuropathy with autonomic features, bilateral carpal tunnel syndrome, or hepatomegaly without imaging abnormalities. Any patient with a monoclonal gammopathy or MM with atypical manifestations such as macroglossia or raccoon eyes. A high index of suspicion is essential to prevent delays in diagnosis. Patients usually presents with one of the above symptoms. Diagnostic testing includes CBC, chemistry panel and heart biomarkers; imaging studies including echocardiogram (heart ultrasound), nuclear medicine technetium pyrophosphate scanning, cardiac MRI and EKG; cardiac and BM biopsy, nerve conduction study and genetic testing (68).

**Mortality.** Cardiac amyloidosis has been associated with a high 5-year mortality of 44–65% after diagnosis (41, 69, 70). Screening efforts have revealed a higher prevalence of hidden CA than previously assumed, indicating that its true burden has been underestimated. Diagnostic tools such as bone tracer scintigraphy, cardiac magnetic resonance imaging, and mass spectrometry, coupled with cutting-edge pharmacotherapy, have led to increased focus on identifying patients with TTR amyloidosis. Consequently, there has been a shift the view of the disease from being rare and untreatable to more common and manageable. Patients with CA often experience significant delays in receiving a proper diagnosis which reduces access to disease-modifying treatments. Also individuals with CA tend to poorly tolerate many medications commonly prescribed for HF and atrial fibrillation. This intolerance can lead to symptom exacerbation and serious adverse reactions. In 2019, Gilstrap et al. (71) provided the first contemporary estimate of cardiac amyloidosis incidence and prevalence among Medicare beneficiaries in the US (48). The incidence of CA was 17/100,000 person-years with a prevalence rate of 55/ 100,000 person-years among hospitalized patients >65 years of age (71). The incidence and prevalence rates of CA among hospitalized patients have increased since 2000, particularly among specific patient subgroups after 2006.

## Current treatments of cardiac amyloidosis

Treatment of CA requires a two-pronged approach, namely to first alleviate cardiac symptoms and complications and second to treat to the underlying condition. Over the past two decades, management of CA has taken advantage of many of the advances of the chemotherapeutic regimens for the plasma cell related malignancy MM (). Key elements in the management of AL amyloidosis are early recognition of the diagnosis, rapid control of the amyloidogenic light chains; and improvement of end organ function.

*Eliminating the plasma cell clone that produces toxic amyloidogenic light chains*. The primary treatment uses chemotherapy and/or immunotherapy to eradicate the aberrant, underlying plasma cell clone and decrease amyloid protein production to thereby limit further organ damage and promote the regression of tissue amyloid deposits (72–77). Management of AL amyloidosis is derived from numerous anti–plasma cell-directed therapies (74–77). The primary aim of therapy in AL amyloidosis is to rapidly eliminate production of the toxic amyloidogenic light chains by targeting the plasma cell clone. Chemotherapy remains the most effective therapy for AL amyloidosis through the elimination of the light-chain–producing plasma cell clone and is based on the adaptation of regimens developed for MM patients. A rapid hematologic response may be critical to optimize response and survival, while the depth of hematologic response is important in order to maximize the probability of organ function improvement and organ response.

*Proteasome inhibitors to target the pathogenic clone cardiac amyloidosis.* Systemic AL amyloidosis is a plasma cell dyscrasia caused by aggregation-prone monoclonal immunoglobulin light chains (LCs) produced by small clones of bone marrow plasma cells (78). The mutated, unstable LCs form insoluble β-pleated sheets that aggregate and deposit systemically in organ tissues as amyloid fibrils causing organ dysfunction and death (39–48). Although pathogenic LCs have been characterized biochemically, until recently little was known about the biology of amyloidogenic plasma cells. Current regimens include proteasome inhibitors and immunomodulatory drugs, e.g., thalidomide and lenalidomide, in combination with alkylating agents. The clinical efficacy of proteasome inhibitors raises the attractive possibility that the amyloidogenic plasma cell clone is a direct target of the proteasome inhibitors. Proteasome inhibitors have shown excellent clinical efficacy with unprecedented response rates, rapidly achieved in both previously untreated and pretreated patients. However, a significant proportion of patients (40%) fail to respond to proteasome inhibitor-based therapy. Bortezomib is a standard therapy in AL amyloidosis, but little is known about response duration (79). A difference in involved amyloidogenic and uninvolved serum-free light chains (dFLC) < 10 mg/L (low dFLC response) predicts survival in AL patients with low presenting dFLC (20–50 mg/L).

The ubiquitin–proteasome system (UPS) plays an important role in the cellular processes for protein quality control and homeostasis (80–82). Cellular proteins are tagged by covalent attachment of the 76 amino acid protein ubiquitin, and targeted for degradation by ATP-dependent 26S proteasomes (80). Ubiquitin is bound to the target protein by an isopeptide linkage between the carboxy terminal glycine of ubiquitin and, usually, the ε-amino group of lysine in the target protein. The proteasome is a highly sophisticated protease complex designed to carry out selective, efficient and processive hydrolysis of client proteins (83–86). Proteasomes function as the catalytic core of the UPS (87, 88). The highly organized proteasome plays a prominent role in the control of a diverse array of basic cellular activities, cell survival and the proliferation of malignant cells (89). Dysregulation of the UPS has been implicated in numerous diseases, including cancer (89–91). Proteasomes are essential, ubiquitous intracellular proteases that degrade a broad variety of cytoplasmic, nuclear, and membrane proteins (92–94). Eukaryotic proteasomes are large protein complexes with a molecular mass ∼2,000 kDa, with a modular architecture (89–91). The catalytic core of the molecule is the 20S proteasome, a cylindrical particle that consists of four heptameric rings made from seven different subunits each, which are present in two copies and in unique locations so that the particle has overall 2-fold symmetry. The proteasome is made up of two subcomplexes: a 20S catalytic core particle (CP; 20S proteasome) and one or two terminal 19S regulatory particle(s) (RP) that serves as a proteasome activator with a molecular mass of approximately 700 kDa (also known as PA700). 20S proteasomes are composed of four heteroheptameric stacked rings in an α7β7β7α7 arrangement. The outer rings consist of α-type subunits whereas the inner two rings consist of β-type subunits. The 19S RP binds to one or both ends of the latent 20S proteasome to form the enzymatically active proteasome.

Proteasome inhibitors have dramatically changed the management of certain hematologic malignancies and significantly improved the progression free survival (PFS) and OS outcomes for patients diagnosed with MM and mantle cell lymphoma (93–96). Two additional proteasome inhibitors, carfilzomib and ixazomib, have been FDA approved and other agents and combinations currently under investigation. Proteasomes degrade ubiquitinated proteins or substrates through the ubiquitin-proteasome pathway, a pathway that is utilized in MM due to the high protein turnover with immunoglobulin production (97, 98). Proteasome inhibitors exploit dependence on this pathway, halting protein degradation that ultimately results in apoptotic cell death. The high protein turnover in MM cells, results in a favorable therapeutic window for proteasome inhibitors in this disease with preferential susceptibility of the malignant cells relative to normal cells. Over the past 20 years, proteasome inhibition has emerged as an effective therapeutic strategy for treating not only MM, but also AL amyloidosis (99–103).

The survival rate of MM patients has improved significantly since clinical introduction of bortezomib and other immunomodulatory drugs (104, 105). However, bortezomib has several limitations. Not all patients respond to bortezomib-based therapies and relapse occurs in many patients who initially responded. Solid tumors, in particular, are often resistant to bortezomib. Furthermore, bortezomib can induce dose-limiting peripheral neuropathy. The second-generation proteasome inhibitor carfilzomib induces responses in a minority of MM patients that relapsed or were refractory to bortezomib. The second-generation proteasome inhibitors, ixazomib, delanzomib, oprozomib and marizomib, each with unique pharmacologic properties and distinct anticancer activities, have demonstrated clinical activity in bortezomib-resistant cancers. Targeting the immunoproteasome, E3 ligases, the 19S RP and deubiquitinases represent additional directions for the generation inhibitors of the UPS (106–110).

An international, multicenter series of 60 patients with Mayo Clinic stage III CA assessed the impact of bortezomib, cyclophosphamide and dexamethasone (VCd) in improving outcomes in this poor-risk group (111, 112). Median follow-up was 11.8 months with an overall response rate of 68%. Patients who survived more than 3 months had an overall response rate of 86% and a cardiac response was seen in 32% of patients. The estimated 1-year survival rate for the whole cohort was 57% and 24 patients (40%) died while on therapy.

Daratumumab (dara) is a high-affinity human IgGκ1 monoclonal antibody that binds to CD38, an antigen ubiquitously expressed all plasma cells, causing cell death by multiple pathways. Dara-containing triplet and quadruplet combinations in MM lead to deep MRD-negative responses with improved PFS and OS (92). Daratumumab was evaluated for newly diagnosed AL amyloidosis patients as front-line treatment of AL amyloidosis. ANDROMEDA was the pivotal phase 3 trial comparing dara-VCd (up to 24 cycles) with VCd alone (6 cycles) in 388 patients with newly diagnosed systemic AL amyloidosis (excluding very advanced disease), with a primary end point of hematologic CR and secondary end points of organ response and MOD-PFS (93). The control group received six cycles of bortezomib, cyclophosphamide, and dexamethasone alone while the dara group received subcutaneous dara followed by single-agent dara every 4 weeks for up to 24 cycles (93). A total of 388 patients underwent randomization and the primary endpoint was a hematologic complete response. The percentage of patients who had a hematologic complete response was significantly higher in the dara group than in the control group (53.3% vs. 18.1%) (RR ratio, 2.9; 95%; CI, 2.1 to 4.1; *p* < 0.001). Survival free from major organ deterioration or hematologic progression favored the dara group (HR for major organ deterioration, hematologic progression, or death, 0.58; 95% CI, 0.36 to 0.93; *p* = 0.02). At 6 months, more cardiac and renal responses occurred in the dara group than in the control group (41.5% vs. 22.2% and 53.0% vs. 23.9%, respectively). Heart failure was reported in 6.2% of the dara group vs 4.8% in the control group. Daratumumab-based regimens achieve deep hematologic and organ responses, offering a new therapeutic backbone. In 2021, the US FDA approved dara, in combination with VCd, as a first-line therapy for newly diagnosed AL amyloidosis following clinical trials that demonstrated rapid hematologic and organ responses.

## New disease-modifying therapies evaluated in clinical trials for ATTR

Significant translational progress in understanding the mechanisms involved in TTR amyloid aggregate formation generated novel, disease-modifying strategies aimed at reducing the deposition of ATTR in the myocardium through stabilization of the circulating TTR tetramer or through a reduction in the hepatic synthesis of TTR.

**1. TTR stabilizers**

*Tafamadis.* ATTR variants are caused by pathogenic single-nucleotide variants in the gene encoding TTR that induce protein misfolding and systemic deposition of amyloid. Tafamadis is a small molecule that stabilizes the circulating TTR tetramer and prevents dissociation in monomers by binding the T4-binding sites. In a recent phase 3, multicenter trial, tafamadis reduced all-cause mortality and cardiovascular hospitalization in patients with HF due to ATTRwt and ATTRv-CA when compared to placebo by 30% (26, 113); Table 2). Tafamadis was administered orally, safe and well tolerated. Patients in NYHA class IV and estimated GFR < 25 ml/min/m^2^ were excluded from the study population and the consistent mortality benefit was evident only after 18–20 months of therapy, predominantly in patients in NYHA I-II. Hence, strict inclusion criteria and careful patient selection was required. Reduced benefit with tafamidis in patients with NYHA functional class III may be related to higher hospitalization rates in this subgroup, potentially because of a longer survival during a more severe phase of the disease.4,9 Tafamidis has become the first disease-modifying drug to be approved for the treatment of ATTRwt and ATTRv-CA. All other drugs are only licensed for the treatment of polyneuropathy caused by ATTR amyloidosis.

**TABLE 2:** Recent trials to evaluate novel therapies to treat transthyretin (ATTR) and associated cardiac amyloidosis and cardiomyopathy.

Trial	Mechanism	Diagnosis	Trial design	Dosing	Adverse events	Primary endpoints
**I. TTR stabilizers**						
NCT01994889 Phase III Tafamadis	Binds to TTR, prevents tetramer dissociation and amyloidogenesis	ATTR amyloid CM	Multicenter International double-blind placebo- controlled 441 patients 2:1:2 ratio	80 mg Tafamadis qd 20 mg Tafamadis qd or placebo for 30 months	Adverse events were similar in the Tafamadis and placebo group. Diarrhea and UTI were previously reported in patients with familial amyloid polyneuropathy were less common in patients who had received Tafamidis than those who received placebo	The all-cause mortality and CV-related hospitalizations were lower among patients who received Tafamadis. At month 30, Tafamadis associated with a lower rate of decline in distance for the 6 min walk test and KCCQ-OS score
NCT03860935 Phase III Acoramidis	High-affinity TTR stabilizer Inhibits the dissociation of tetrameric TTR	TTR Cardiac Amyloid CM	Double-blind randomized 632 patients 2:1 ratio	800 mg acoramidis bid vs. placebo for 30 months	The overall incidence of AEs was similar in the acoramidis group and the placebo group	During the 30 month trial period, the four-step primary hierarchical analysis included death from any cause, frequency of cardio- vascular cumulative-related hospitalization, the change from baseline in the NT-pro-BNP level, and the change from baseline in the 6 min walk distance was significantly better in the Acoramidis group than the placebo group.
**II. Inhibitors of TTR gene expression**						
NCT03997383 Phase III Patisiran	RNA interference therapeutic that inhibits production of hepatic TTR	Hereditary (variant) ATTR Cardiac Amyloidosis	Double-blind randomized 360 patients 1:1 ratio	0.3 mg/kg BW or placebo q 3 weeks for 12 months	Mild-moderate AEs were infusion-related reactions, arthralgia, and muscle spasms. Serious AEs in the Patisiran vs. placebo groups were cardiac failure, atrial fibrillations, complete AV block, amyloidosis and syncope.	The magnitude of decline from baseline in the 6 min walk distance at 12 months after starting Patisiran was significantly lower in the Patisiran group than the placebo group with a median change from baseline of −8.15 m.
NCT01737398 Phase III Inotersen	Antisense Oligonucleotide inhibits hepatic production of TTR	Hereditary ATTR	Randomized double-blind placebo-controlled adults with stage1 or 2 hereditary ATTR with poly- neuropathy172 patient 2:1 ratio 112- Inotersen group 60- placebo group	Weekly sc injection of Inotersen (300mg) or placebo	Five deaths in total in the Inotersen group Biopsy-confirmed glomerulonephritis in three patients. One fatal intracranial hemorrhage associated with low plt. count. AEs included nausea, vomiting, chills, low platelets, pyrexia, anemia.	Change in the modified neuropathy impairment score+7 (mNIS+7) and the Norfolk QOL-DN score from baseline to week 66 after 15 months of intervention with Inotersen.
NCT02319005 Phase III Revusiran (Endeavor)	Gal-NAc conjugated siRNA	ATTRv-CA	Multicenter, international, randomized double-blind, placebo- controlled Phase 3 study	SC 500 mg (*n* = 140) or placebo (*n* = 66) followed by weekly doses	Study was discontinued because of an imbalance in mortality observed between	Co-primary end points were 6 min walk test distance and serum TTR reduction (125)
					Revusiran treatment stopped after a median of 6.71 months, the study sponsor prematurely discontinued dosing due to an observed mortality imbalance between the treatment arms. Eighteen (12.9%) on revusiran and 2 (3%) on placebo during the on treatment period. Most deaths in both treatment arms were indicated as cardiovascular due to HF.	
NCT04153149 HELIOS-B trial Phase 3	RNA inhibitor	Variant or wt ATTR CM	Multicenter, double-blind, randomized, placebo- controlled trial, Patients with total of 655 patients underwent randomization; 326 were assigned to receive vutrisiran and 329 to receive placebo	SC 25 mg (*n* = 326) placebo (*n* = 329) q 12 weeks, up to 36 months	AEs was similar in the vutrisiran group 99% of and placebo (98%). Serious adverse events occurred in 62% of the vutrisiran group and 67% of the placebo group which led to the discontinuation in3% of the vutrisiran and 4% of placebo group.	The primary end was a composite of death from any cause and recurrent CV events. Vutrisiran treatment led to a lower risk of death and CV events than placebo
**III. Recombinant anti-TTR antibody**						
NCT04360434 Phase I NI006 Antibody	Recombinant hu anti-ATTR Ab removes ATTR by phagocytic immune cells	WT or variant ATTR CM and CHF	Double-blind 40 patients 2:1 ratio	IV infusion or placebo q 4 weeks for 4 months. Dosing ranged from 0.3 to 60 mg/kg BW	No apparent, drug-related SAEs were reported	Safety and PK profiles of NI006 were assessed and cardiac imaging performed. PK profile of NI006 was consistent with that of an IgG Ab. No anti-drug Abs were detected at doses >10 mg/kg BW, cardiac tracer uptake scintigraphy and extracellular volume on cardiac MRI, both of which are imaging-based, surrogate markers of cardiac amyloid load, appeared to be reduced over 12 months. Exposure to >10 mg/kg appeared to reduce the median N-terminal pro-BNP and troponin T seemed reduced.

TTR, transthyretin; ATTR, transthyretin amyloid; WT, wildtype; CM, cardiomyopathy; BW, body weight; UTI, urinary tract infection; Plt, platelet; QOL DN, Norfolk Quality of life-diabetic neuropathy; CV, cardiovascular; KCCQ, Kansas City Cardiomyopathy Questionnaire; Hu, human; PK, pharmacokinetics; CHF, congestive heart failure; NT, N-terminal; Ab, antibody; AE, adverse effect; SAE, serious adverse event; AV, atrioventricular; BNP, Pro-B-type natriuretic peptide.

*Acoramadis.* Acoramidis is a high-affinity TTR stabilizer that acts to inhibit dissociation of tetrameric TTR and leads to more than 90% stabilization across the dosing interval as measured *ex vivo* (26, 114). Acoramidis is a next generation TTR stabilizer that mimics the structural stabilizing properties of the TTR variant p.T139M. The affinity and potency of acoramidis appears to exceed that of tafamidis resulting in a more effective stabilization of circulating TTR, both wild-type and variant. Patients (*n* = 632) underwent randomization and the primary analysis favored acoramidis over placebo (*p* < 0.001, win ratio was 1.8 with 95% CI, 1.4–2.2). Acoramidis resulted in a significantly better four-step primary hierarchical outcome assessed by mortality, morbidity, and function than placebo.

**2. Inhibitors of *TTR* gene expression**

*Patirisan.* Patirisan is a lipid nanoparticle encapsulated siRNA that inhibits the expression of TTR (both WT and variant) in the hepatocytes by disrupting the *TTR* mRNA (115–117). In the phase 3 multicenter APOLLO trial on patients with polyneuropathy caused by ATTRv amyloidosis, patisiran administered IV (0.3 mg/kg once every 3 weeks for 18 months) significantly improved neurological status. Echocardiography was consistent with ATTRv-CA in 56% of patients enrolled in the study. Patisiran promoted favorable myocardial remodeling, by reducing mean LV wall thickness, relative wall thickness and serum NT-proBNP levels. In October 2023, the US FDA declined to approve patirisan (Onpattro, Alnylam Pharmaceuticals) for treatment of ATTR amyloidosis with cardiomyopathy.

*Inotersen.* Hereditary ATTR amyloidosis is caused by pathogenic single-nucleotide variants in the *TTR* gene that induce TTR misfolding and systemic deposition of amyloid. Inotersen, a 2′- O-methoxyethyl-modified antisense oligonucleotide, inhibits hepatic production of transthyretin. TR is synthesized in liver and secreted into the blood and Inotersen (also known as ISIS 420915), a 2′-*O*-methoxyethyl–modified antisense oligonucleotide, inhibits hepatic production of TTR (66, 118). Inotersen was designed to decrease the amount of mutant and normal TTR made by the liver. The agent was predicted to decrease the amount of TTR protein and result in the decreased formation of TTR deposits, to slow or stop disease progression. An international, randomized, phase 3 trial of inotersen in patients with stage 1 (ambulatory patient) or stage 2 (ambulatory with assistance) hATTR with polyneuropathy randomly assigned patients to receive weekly injections of inotersen or placebo. Primary endpoints were the change in the modified Neuropathy Impairment Score+7 (mNIS+7; range, −22.3 to 346.3, with higher scores indicating poorer function) and the change in the score on the patient-reported Norfolk Quality of Life–Diabetic Neuropathy (QOL-DN) questionnaire.

Glomerulonephritis and severe thrombocytopenia were reported in 3% of patients receiving inotersen and one patient died from intracranial hemorrhage related to thrombocytopenia. In the open-label extension with enhanced monitoring, the rate of thrombocytopenia was similar among inotersen group and placebo group and there were no cases of severe thrombocytopenia or acute glomerulonephritis. Nevertheless, patients on inotersen require regular monitoring.

**3. Recombinant anti-TTR antibody**

*NI006.* NI006 is a recombinant human anti-ATTR antibody developed to remove ATTR by phagocytic immune cells (119). A phase 1, double-blind trial, randomly assigned (in a 2:1 ratio) 40 patients with wt or variant ATTR cardiomyopathy and CHF to receive NI006 or placebo every 4 weeks for 4 months. Patients were sequentially enrolled in six cohorts that received ascending doses (ranging from 0.3 to 60 mg/kg BW). After four infusions, patients were enrolled in an open-label extension phase in which they received eight infusions of NI006 with stepwise increases in dose. At doses of at least 10 mg/ kg, cardiac tracer uptake on scintigraphy and extracellular volume on cardiac MRI, both of which are imaging-based surrogate markers of cardiac amyloid load, appeared reduced over a 12 months. Median N-terminal pro–B-type natriuretic peptide and troponin T levels also appeared reduced.

*Revusiran.* The Phase 3 ENDEAVOR study evaluated the investigational RNA interference therapeutic revusiran, that targets hepatic TTR production, for the treatment of CA caused by hereditary transthyretin-mediated amyloidosis, Patients with CA were randomized 2:1 to receive subcutaneous daily revusiran 500 mg (*n* = 140) or placebo (*n* = 66) for 5 days over a week followed by weekly doses. Co-primary endpoints were 6-min walk test distance and serum TTR reduction. However, revusiran treatment was stopped after a median of 6.71 months; the study Sponsor prematurely discontinued dosing due to an observed mortality imbalance between treatment arms. Eighteen (12.9%) patients on revusiran and 2 (3.0%) on placebo died during the on-treatment period. Most deaths in both treatment arms were adjudicated as cardiovascular due to HF. A *post hoc* safety investigation of patients treated with revusiran found that, at baseline, a greater proportion of those who died were ≥75 years and showed clinical evidence of more advanced HF compared with those who were alive throughout treatment. Revusiran pharmacokinetic exposures and TTR lowering did not show meaningful differences between patients who died and who were alive. Revusiran did not deleteriously affect echocardiographic parameters, cardiac biomarkers, or frequency of cardiovascular and HF hospitalization events. A clear causative mechanism could be identified as a cause for the observed mortality imbalance associated with revusiran.

*Vutrisiran.* Vutrisiran is a subcutaneously administered RNA interference therapeutic that inhibits the production of hepatic TTR (120). A double-blind, randomized trial, we assigned patients with ATTR-CM in a 1:1 ratio to receive vutrisiran (25 mg) or placebo every 12 weeks for up to 36 months. The primary end point was a composite of death from any cause and recurrent cardiovascular events. Secondary end points included death from any cause, the change from baseline in the distance covered on the 6-min walk test, and the change from baseline in the Kansas City Cardiomyopathy Questionnaire–Overall Summary score. A total of 655 patients underwent randomization; 326 were assigned to receive vutrisiran and 329 to receive placebo. Vutrisiran treatment led to a lower risk of death from any cause and recurrent cardiovascular events than placebo (HR in the overall population, 0.72; 95% CI, 0.56 to 0.93; *P* = 0.01; HR in the monotherapy population, 0.67; 95% CI, 0.49 to 0.93; *P* = 0.02) and a lower risk of death from any cause through 42 months (HRin the overall population, 0.65; 95% CI, 0.46 to 0.90; *P* = 0.01). Among the patients in the overall population, 125 in the vutrisiran group and 159 in the placebo group had at least one primary end-point event. In the overall population, treatment with vutrisiran resulted in less of a decline in the distance covered on the 6-min walk test than placebo (least-squares mean difference, 26.5 m; 95% CI, 13.4 to 39.6; *P* < 0.001) and less of a decline in the KCCQ-OS score (least-squares mean difference, 5.8 points; 95% CI, 2.4 to 9.2; *P* < 0.001).

Treatment with vutrisiran led to a lower risk of death from any cause and cardiovascular events than placebo and preserved functional capacity and quality of life.

## Conclusions and relevance

Over the past decade, the care of patients diagnosed with CA has undergone transformative changes, leading to the marked, steady progress in outcomes for patients (121–125). Cardiac transplantation previously had been performed in patients with CA, but outcomes were not favorable secondary to extra-cardiac disease involvement and limitations of chemotherapy options. With the advent of plasma cell-directed and disease-modifying therapeutics, proper candidates can be selected for organ transplant. The management of CA is expected to further evolve and improve with a better understanding of the specific mechanisms of amyloidogenesis and myocardial deposition. There is increasing interest in disease-modifying treatment with the development of TTR stabilizers, gene silencers, therapeutic antibodies and the targeted delivery of gene-editing therapeutics aimed at directly eradicating amyloid deposits. ATTR amyloidosis causes cardiomyopathy in up to 150,000 people in the US and tafamidis is the only currently approved therapy. Tafamidis slowed progression of ATTR amyloidosis and improved survival and prevented hospitalization, compared with placebo, in patients with ATTR-associated cardiomyopathy.

**Limitations.** This review may be limited since some evidence was based on relatively small studies or retrospective studies that may have been influenced by selection and/or referral bias. Second, studies may have been missed.
